# Resistance Landscape and Clonal Dynamics of ESKAPE Pathogens in Bloodstream Infections: A Multicenter Study from Mexico

**DOI:** 10.3390/pathogens14111187

**Published:** 2025-11-19

**Authors:** María Dolores Alcántar-Curiel, Rayo Morfín-Otero, Ma Dolores Jarillo-Quijada, José Luis Fernández-Vázquez, Catalina Gayosso-Vázquez, María Luisa Hernández-Medel, Manuelita Zavala-Pineda, Miguel Ángel Morales-Gil, Mónica Osorio-Guzmán, María Angelina Quevedo-Ramos, Luis Fernando Pérez-González, Andrés Flores-Santos, Sergio Esparza-Ahumada, Rodrigo Escobedo-Sánchez, Roberto Rosales-Reyes, José Eduardo Toledano-Tableros, Silvia Giono-Cerezo, José Ignacio Santos-Preciado, Eduardo Rodríguez-Noriega

**Affiliations:** 1 Unidad de Investigación en Medicina Experimental, Facultad de Medicina, Universidad Nacional Autónoma de México, Ciudad de México 04510, Mexico; mdjarilloq@comunidad.unam.mx (M.D.J.-Q.); joseluisfev@gmail.com (J.L.F.-V.); catalina_gayosso@yahoo.com.mx (C.G.-V.); rrosalesr@comunidad.unam.mx (R.R.-R.); neptuno_jett@hotmail.com (J.E.T.-T.); joseignaciosantos56@gmail.com (J.I.S.-P.); 2 Hospital Civil de Guadalajara Fray Antonio Alcalde, Guadalajara Jalisco 44280, Mexico; rayomorfin@gmail.com (R.M.-O.); checo.esparza@gmail.com (S.E.-A.); wankov1995@gmail.com (R.E.-S.); idfcolima@yahoo.com (E.R.-N.); 3 Instituto de Patología Infecciosa y Experimental “Dr. Francisco Ruiz Sánchez”, Centro Universitario de Ciencias de la Salud, Universidad de Guadalajara, Guadalajara Jalisco 45129, Mexico; 4 Unidad de Infectología y Laboratorio Central de Microbiología, Hospital General de México Eduardo Liceaga, Ciudad de México 06720, Mexico; infectologiahgm@gmail.com (M.L.H.-M.); mzavala_pineda@hotmail.com (M.Z.-P.); yemivan04@gmail.com (M.Á.M.-G.); 5 Hospital General de León, León Guanajuato 37250, Mexico; monica.osorio.guzman@gmail.com (M.O.-G.); maquevedor@hotmail.com (M.A.Q.-R.); 6 Hospital Central Dr. Ignacio Morones Prieto, San Luis Potosí 78240, Mexico; luisfer.luisfernando@gmail.com (L.F.P.-G.); andres.santos@uaslp.mx (A.F.-S.); 7 Escuela Nacional de Ciencias Biológicas, Instituto Politécnico Nacional, Ciudad de México 11340, Mexico; sgiono@yahoo.com

**Keywords:** antimicrobial resistance, bacteremia, bloodstream infections, clonal dissemination, ESKAPE bacteria, Mexico

## Abstract

Antimicrobial resistance in healthcare-associated infections represents one of the greatest threats to global health. The COVID-19 pandemic disrupted infection control and antimicrobial stewardship, potentially affecting the prevalence of pathogens and the development of resistance. This study aimed to investigate the prevalence, antimicrobial resistance, and clonal dissemination of ESKAPE pathogens isolated from bloodstream infections during the second year of the COVID-19 pandemic in four tertiary-care hospitals in Mexico. A total of 926 isolates were analyzed: *Staphylococcus aureus* (22.4%), *Klebsiella pneumoniae* (22%), *Acinetobacter baumannii* (21.5%), *Pseudomonas aeruginosa* (12.5%), *Enterobacter cloacae* (9.4%), *Enterococcus faecalis* (8.4%), and *Enterococcus faecium* (3.8%). High rates of multidrug resistance were observed in *A. baumannii* (70.9% XDR) and *K. pneumoniae* (71% XDR plus MDR with 79% ESBL). *P. aeruginosa* and *E. cloacae* showed the highest susceptibility rates (53% and 48%, respectively) to all antimicrobials. The main β-lactamases involved in resistance were *bla*_SHV_, *bla*_CTX-M_, and *bla*_TEM_ in *K. pneumoniae,* while the predominant carbapenemases were *bla*_OXA-24_, *bla_OXA-23_ in A. baumannii, bla_NDM_* in *K. pneumoniae,* and *bla*_VIM_ in *P. aeruginosa.* Among Gram-positives, methicillin-resistant *S. aureus* accounted for 33.8% of isolates, and vancomycin resistance was higher in *E. faecium* (28%) than in *E. faecalis* (1.3%). Pulsed-field gel electrophoresis revealed endemic circulation of *A. baumannii* clones (Pulsotypes 1AC, 2AM), persistent for over a decade, and interhospital dissemination of *S. aureus* and *K. pneumoniae* clones. These findings underscore the epidemiological relevance of MDR ESKAPE pathogens during the COVID-19 pandemic and highlight the urgent need to optimize empirical therapy and maintain continuous genomic surveillance to enhance infection control in Mexican hospitals.

## 1. Introduction

The global spread of antimicrobial-resistant (AMR) pathogens, especially within healthcare-associated infections (HAIs), truly creates a serious challenge for healthcare systems [1]. In 2019, around 569,000 deaths were associated with AMR, including 141,000 direct deaths, which came out of the 35 countries of the WHO Region of Americas [2]. HAIs are primarily caused by bacteria that exhibit a remarkable ability to resist the effects of antibiotics and express multiple virulence mechanisms. Among the most concerning multidrug-resistant (MDR) bacteria are the ESKAPE pathogens—*Enterococcus faecium*, *Staphylococcus aureus*, *Klebsiella pneumoniae*, *Acinetobacter baumannii*, *Pseudomonas aeruginosa*, and *Enterobacter* spp.—which are responsible for a considerable proportion of bloodstream infections, increasing morbidity, mortality, hospital stays, and healthcare costs, particularly among immunocompromised patients [3,4]. Globally recognized health authorities, like the WHO, have emphasized how vital surveillance is for AMR, moreover spotlighting ESKAPE pathogens, as critical priority organisms to prioritize in research and in the development of new therapeutic strategies [5,6]. Such concern mainly originates from these pathogens' resistance to last-resort antibiotics, such as carbapenems, and moreover from their ability to clonally disseminate and persist in hospital environments.

Mexico is a country characterized by marked geographic and socioeconomic diversity, and it shows considerable regional variability in the AMR landscape. MDR ESKAPE pathogens have been reported in both public and private healthcare facilities, as evidenced by national surveillance efforts [7,8,9]. However, comprehensive multicenter studies incorporating genetic characterization remain scarce.

Clonal dissemination and genetic profiling are key to understanding the epidemiology and evolution of antimicrobial resistance in ESKAPE pathogens. Their ability to spread through dominant clones carrying transferable resistance and virulence genes enables persistence and rapid expansion in hospitals [10]. Molecular typing methods, such as PFGE and PCR detection of resistance genes, are crucial for identifying outbreaks and high-risk clones [11]. Combining phenotypic resistance data with genetic and clonal analyses provides a comprehensive view of pathogen adaptation and dissemination in healthcare settings [10,11,12].

The COVID-19 pandemic further underscored the impact of antibiotic overuse and deficiencies in infection control practices, which contributed to an increase in HAIs [13]. Indeed, hospitals in Monterrey and Mexico City have reported increased incidences of MDR pathogens exacerbated by the pandemic [14].

This study aimed to determine the frequency, antimicrobial resistance patterns, and genetic profiles of ESKAPE pathogens isolated from bloodstream infections in four tertiary-care hospitals across Mexico during the COVID-19 pandemic, providing the first multicenter study in the country that integrates phenotypic resistance, genetic determinants, and clonal dissemination analyses of these pathogens. Our findings provide critical evidence of the high prevalence of MDR and clonal spread, underscoring the need for continued molecular surveillance, reinforced infection control measures, and alignment with global initiatives such as the WHO Global Antimicrobial Resistance and Use Surveillance system Report (GLASS) to mitigate the dissemination of these highly resistant pathogens [6]. The data generated in this study aims to improve clinical decision-making, support infection control programs, and guide national AMR surveillance policies in Mexico.

## 2. Materials and Methods

### 2.1. Clinical Setting

This study was conducted in four tertiary referral hospitals in different geographic regions of Mexico. The Hospital General de México “Dr. Eduardo Liceaga” (HGM), located in Mexico City, has more than 1000 beds and provides care for populations without access to public health services in the metropolitan area and in states lacking highly specialized facilities.; the Hospital Civil de Guadalajara “Fray Antonio Alcalde” (HCG) in Guadalajara, Jalisco, a large university hospital with more than 900 beds, primarily serves the population of western Mexico; the Hospital General de León (HGL) in León, Guanajuato, a secondary and tertiary care hospital with approximately 300 beds, covers the Bajío region, and the Hospital Central “Dr. Ignacio Morones Prieto” in San Luis Potosí, San Luis Potosí (HSLP), with 250 beds, is a referral hospital providing highly specialized care for populations in the north-central region of Mexico. Clinical isolates and their antimicrobial susceptibility profiles were identified in the microbiology laboratories of each hospital.

### 2.2. Bacterial Isolation

All isolates of *E. faecium*, *E. faecalis*, *S. aureus*, *K. pneumoniae*, *A. baumannii*, *P. aeruginosa*, and *E. cloacae* were obtained from blood cultures of hospitalized patients with bloodstream infections classified as HAIs, defined by physicians from the Infectious Diseases Unit according to the criteria published by the Centers for Disease Control and Prevention (CDC) [15]. Non-duplicate clinical isolates collected between January and December 2021 were included in this study. Samples were managed anonymously throughout the process to ensure patient confidentiality. For the purposes of this investigation, one isolate per patient, per bacterial species, and per infection episode was considered non-duplicate. Only the first isolate of a given species obtained from the same patient and infection episode was included in the analysis. Subsequent isolates were excluded unless they exhibited a different antimicrobial susceptibility profile or corresponded to a new infection episode.

Blood samples were obtained under aseptic conditions using iodine and 70% ethanol for skin disinfection, and approximately 10 mL of blood was inoculated into Bact-Alert^®^ blood culture bottles (bioMérieux, Marcy-l’Étoile, France). The bottles that were inoculated with blood were incubated for 5 days in the Bact-Alert^®^ system. After bacterial growth was observed, 3 mL of broth was aseptically withdrawn from each sample, and one drop of broth was streaked onto 5% sheep blood agar, MacConkey agar, and Sabouraud agar plates for isolation of colonies. Plates were incubated at 37 °C for 24 h. If mixed growth was noted, then colonies were subcultured to obtain pure colonies. A single colony was suspended in 3 mL of sterile 0.75% saline for a turbidity of 0.5 McFarland standard. This standardized suspension was used for bacterial identification and antimicrobial susceptibility testing with the automated VITEK^®^ 2 system (bioMérieux, Marcy-l’Étoile, France). Isolates were stored in Luria–Bertani broth (Difco, BD Biosciences, Franklin Lakes, NJ, USA) supplemented with 20% glycerol (Sigma-Aldrich, St. Louis, MO, USA) at −70 °C until further analysis.

### 2.3. Antimicrobial Susceptibility Testing

Antimicrobial susceptibility testing was performed using the automated VITEK^®^-2 System (bioMérieux, Marcy-l’Étoile, France). The antimicrobial minimum inhibitory concentration (MIC) was performed using two methods depending on the antibiotic evaluated; for most antimicrobial testing, we used serial 2-fold agar dilution in Mueller–Hinton agar (Becton Dickinson, Franklin Lakes, NJ, USA) with final antibiotic concentrations equal to that of the commercial product, ranging from 0.004 to 128 µg/mL. Bacterial suspensions were prepared to 0.5 McFarland, diluted 1:10 in sterile saline, and inoculated onto agar plates using a Steers replicator. The agar plates were incubated at 37 °C for 24 h under Clinical and Laboratory Standards Institute (CLSI) guidelines [16]. The MIC was recorded as the lowest concentration of antibiotic that prevented visible growth. For colistin (Sigma-Aldrich, St. Louis, MO, USA), MICs were performed using the broth microdilution method under CLSI and the European Committee on Antimicrobial Susceptibility Testing (EUCAST) [17] methodology. Stock solutions for colistimethate sodium (Sigma-Aldrich, St. Louis, MO, USA) were prepared in cation-adjusted Mueller–Hinton broth (CAMHB; Becton Dickinson, NJ, USA) and diluted to final concentrations of 0.06–64 µg/mL. Microplates (96 wells) were inoculated with standardized bacterial suspensions (0.5 McFarland) and incubated at 37 °C for 24 h before visual reading of bacterial growth. Results were interpreted using CLSI [16] and EUCAST [17] guidelines. *Escherichia coli* ATCC 25922 and *S. aureus* ATCC 25923 were used as quality control strains. Isolates were classified as MDR, extensively drug-resistant (XDR), or pandrug-resistant (PDR) according to the definitions proposed by Magiorakos et al. [18]. Based on these criteria, MDR isolates were defined as those resistant to at least one agent in three or more antimicrobial categories; XDR as those resistant to all but two or fewer categories; and PDR as those resistant to all agents in all antimicrobial categories tested.

Extended-spectrum β-lactamases (ESBL) production was confirmed by the CLSI-recommended combined disk method using ceftazidime (30 µg) (Sigma-Aldrich, St. Louis, MO, USA) and cefotaxime (30 µg) (Sigma-Aldrich, St. Louis, MO, USA), with and without clavulanic acid (10 µg) (SmithKline Beecham Pharmaceuticals, Worthing, UK). Carbapenemase production was determined by the modified carbapenem inactivation method (mCIM), and metallo-β-lactamases (MBL) production was confirmed by the EDTA-modified carbapenem inactivation method (eCIM) following CLSI guidelines [16]. *Escherichia coli* ATCC 25922 and *K. pneumoniae* ATCC 700603 were used as quality control strains for ESBL production, while *K. pneumoniae* ATCC BAA-1705 was used as a quality control strain for the carbapenemase production test.

For the Gram-positive bacteria, the following test were performed: in *S. aureus* β-lactamase production was assessed using nitrocefin test (Becton Dickinson, Sparks, MD, USA), methicillin resistance was determined by cefoxitin (30 µg) test (Becton Dickinson, Sparks, MD, USA) disk diffusion, and clindamycin test (Becton Dickinson, Sparks, MD, USA) resistance was evaluated using the D-zone test. For *Enterococcus* spp., high-level aminoglycoside resistance test (HLAR) was determined by disk diffusion with gentamicin (120 µg) (Becton Dickinson, Sparks, MD, USA) and streptomycin (300 µg) (Becton Dickinson, Sparks, MD, USA) following CLSI guidelines [16]. The following quality control strains were included: *S. aureus* ATCC 25923, *S. aureus* ATCC 43300, *E. faecalis* 29212, and *E. faecalis* ATCC 51299 [16].

### 2.4. Genetic Identification of Resistance Genes in ESKAPE Bacteria

The presence of resistance genes was screened by PCR. Total DNA extraction for endpoint PCR assays was performed by heat lysis. Briefly, a single colony grown overnight on solid medium was suspended in 20 µL of water, heated at 95 °C for 5 min, and centrifuged; 3 µL of the supernatant were used as a template for each PCR reaction. PCR reactions were carried out in a final volume of 20 µL using the GoTaq^®^ Green Master Mix kit (Promega, Madison, WI, USA) at a final concentration of 1X, with 10 mM of each primer and <250 ng of template DNA, under the amplification conditions previously described (Table 1). This reaction mixture was also used for multiplex PCR assays targeting the *bla_VIM_*, *bla_IMP_*, *bla_NDM_*, *bla_OXA-24_*, and *bla_KPC_* genes by adding the corresponding primer pairs to the amplification reaction. Amplified products were visualized by electrophoresis on 1% agarose gels at 120 V using a Power Station 300 chamber. Amplicons were purified using the Zymoclean™ Gel DNA Recovery Kit (Zymo Research, Irvine, CA, USA) according to the manufacturer’s instructions and sequenced by the Sanger method at the Institute of Biotechnology, National Autonomous University of Mexico. Nucleotide sequences were verified for quality and analyzed using the BLASTx v2.17.0 program available at the National Center for Biotechnology Information (http://www.ncbi.nlm.nih.gov/blast, accessed on 20 may 2025.) with default parameters.

Gram-negative isolates were tested for ESBL genes (*bla*_TEM_, *bla*_CTX__-M_, and *bla*_SHV_), MBL genes (*bla*_VIM_, *bla*_IMP,_ and *bla*_NDM_), OXA-type carbapenemase genes (*bla*_OXA__-23_, *bla*_OXA__-24_, *bla*_OXA__-48_, and *bla*_OXA__-58_), and the serin carbapenemase *bla*_KPC_ gene using specific primers (Table 1). Gram-positive isolates were assessed for the presence of the v*anA* gene associated with vancomycin resistance, *the blaZ* gene associated with penicillin resistance, and the *mecA* gene in *S. aureus* for methicillin resistance. Additionally, aminoglycoside-modifying enzyme genes *(aac(6′)-Ie-aph(2″)-Ia, aph(3′)-IIIa, ant(4′)-Ia,* and erythromycin ribosome methylase genes *(ermA, ermB, ermC,* and *msrA)* for macrolide resistance. In *Enterococcus* spp., the *vanA* gene for vancomycin resistance was also sought, together with aminoglycoside-modifying enzyme genes *acc(6′)-Ie-aph(2″)-Ia, aph(3′)-III, acc(6′-aph(2),* and *aph(2″)-Ic,* the *ermB* gene for macrolide resistance, and the *tetM* gene for tetracycline resistance, using specific primers (Table 1).

### 2.5. Pulsed-Field Gel Electrophoresis (PFGE)

Genotyping of isolates was performed by PFGE as previously described [12,29,30]. Briefly, samples were embedded in 1% agarose plugs, and genomic DNA was digested overnight with *Apa*I (New England Biolabs, Beverly, MA, USA) for *A. baumannii*, *Bcu*I (Invitrogen, now Thermo Fisher Scientific, Waltham, Massachusetts, USA) for *P. aeruginosa*, and *Xba*I (Invitrogen, now Thermo Fisher Scientific) for *E. cloacae* and *K. pneumoniae* at 25 °C, 37 °C, and 37 °C, respectively. For *S. aureus*, *E. faecium*, and *E. faecalis*, DNA was digested overnight at 37 °C with *Sma*I (Invitrogen, now Thermo Fisher Scientific) according to the manufacturer’s instructions. The digested DNA samples were subjected to PFGE using a GenePath system (Bio-Rad^®^, Hercules, CA, USA) with the Gene Path Program Upgrade Kit Version 1.0 (DOS-based software). The following electrical configuration was used: PSU program (*Pseudomonas aeruginosa* and *Acinetobacter baumannii*): initial switch time 5.3 s, final switch time 34.9 s, linear ramping, voltage gradient 6.0 V/cm, included angle 120°, and run time 19.5 h. Eco program (*Klebsiella pneumoniae* and *Enterobacter cloacae*): initial switch time 5.3 s, final switch time 49.9 s, linear ramping, voltage gradient 6.0 V/cm, included angle 120°, and run time: 20 h. StA program (*Staphylococcus aureus*, *Enterococcus faecium,* and *Enterococcus faecalis*): initial switch time 5.3 s, final switch time 34.9 s, linear ramping, voltage density 6.0 V/cm, included angle 120°, and run time 20 h. Pulsotype (PT) was assigned based on the electrophoretic profiles of the isolates according to Tenover’s criteria [31]. The similarity between profiles was calculated from a binary matrix of band presence/absence, using the unweighted pair group method with arithmetic mean (UPGMA) and the Dice coefficient [32]. A similarity of 85% was considered indicative of belonging to the same PT. Clonal dissemination over time and potential outbreaks were also evaluated. PTs were designated using the ordinal number assigned to each PT according to the date of detection, followed by the initial of the microorganism genus (“E” for *Enterobacter*, “S” for *Staphylococcus*, “K” for *Klebsiella*, “A” for *Acinetobacter*, “P” for *Pseudomonas*, “Efa” for *E. faecium*, and “Efe” for *E. faecalis*), and a capital letter corresponding to each hospital (“C” for HCG, “M” for HGM, “L” for HGL, and “P” for HSLP).

### 2.6. Statistical Analysis

The data were analyzed using descriptive statistics to summarize the frequency and antimicrobial resistance patterns of ESKAPE isolates in each hospital and clonal dissemination between hospitals.

## 3. Results

### 3.1. Clinical Isolates of ESKAPE Pathogens

A total of 926 non-duplicate isolates causing bacteremia were collected from 926 individual patients across the four participating hospitals. The HCG contributed the most considerable number of isolates (*n* = 578), followed by the HGM (*n* = 214), HGL (*n* = 93), and HSLP (*n* = 41). Overall, 65.4% of isolates were Gram-negative, of which 48% corresponded to *Enterobacterales* (*K. pneumoniae n* = 204; *E. cloacae n* = 87) and 52% to non-fermenting bacteria (*A. baumannii n* = 199; *P. aeruginosa n* = 116), according to lactose fermentation on MacConkey agar. The remaining 34.6% were Gram-positive, comprising *S. aureus* (*n* = 207, 64.7%), *E. faecalis* (*n* = 78, 24.4%), and *E. faecium* (*n* = 35, 10.9%). Regarding the distribution of ESKAPE pathogens, *S. aureus* was the most frequent species (22.4%), followed by *K. pneumoniae* (22%), *A. baumannii* (21.5%), *P. aeruginosa* (12.5%), *E. cloacae* (9.4%), *E. faecalis* (8.4%), and *E. faecium* (3.8%).

### 3.2. Antimicrobial Susceptibility in the ESKAPE Pathogens

**Figure 1:**
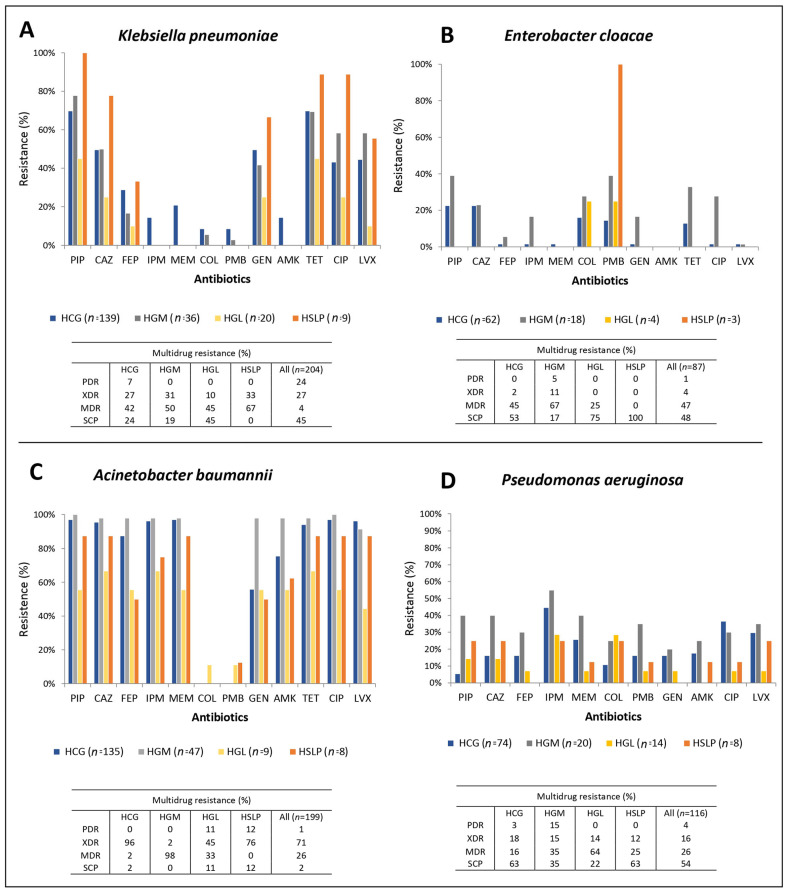
Antimicrobial resistance rates and multidrug resistance in Gram-negative isolates from four hospitals in Mexico. (**A**) *Klebsiella pneumoniae*, (**B**) *Enterobacter cloacae*, (**C**) *Acinetobacter baumannii*, (**D**) *Pseudomonas aeruginosa*. PIP: piperacillin, CAZ: ceftazidime, FEP: cefepime, IPM: imipenem, MEM: meropenem, COL: colistin, PMB: polymyxin B, GEN: gentamicin, AMK: amikacin, TET: tetracycline, CIP: ciprofloxacin, LVX: levofloxacin, PDR: pandrug-resistant, XDR: extensively drug-resistant, MDR: multidrug-resistant, SCP: susceptible. HCG, Hospital Civil de Guadalajara “Fray Antonio Alcalde” in Guadalajara, Jalisco; HGM, Hospital General de México “Dr. Eduardo Liceaga” in Mexico City; HGL, Hospital General de León in León, Guanajuato; and HSLP, Hospital Central “Dr. Ignacio Morones Prieto” in San Luis Potosí.

Based on CLSI guidelines, the antimicrobial susceptibility of all ESKAPE isolates collected in this study was determined. The resistance rates for Gram-negative bacteria across the four hospitals are summarized in Figure 1. Among the Gram-negative pathogens, *A. baumannii* exhibited the highest resistance rates, close to 95.5%, to almost all antibiotics, including resistance to carbapenems in HCG and HGM. Resistance to polymyxin ranged from 0.5% to 1%. *K. pneumoniae* showed resistance rates of 48.5% to third-generation and 25% to fourth-generation cephalosporins, with carbapenem resistance detected in only one hospital, reaching 14%. In *P. aeruginosa*, carbapenem resistance was as high as 43% for IPM and 25% for MEM, while resistance to COL was 16% and to PMB was 18%. *E. cloacae* displayed the lowest resistance rates to most antibiotics, with 1.1% for AMK and 4.6% for carbapenem.

Regarding multidrug resistance categories, *A. baumannii* and *P. aeruginosa* exhibited the highest PDR rates (4.5% and 4.3%, respectively). *A. baumannii* also showed the highest rates of XDR isolates (70.9%), while *E. cloacae* presented the highest proportion of MDR isolates (47.2%). Overall, the highest susceptibility rates were observed in *P. aeruginosa* (53%) and *E. cloacae* (48%) (Figure 1).

**Figure 2:**
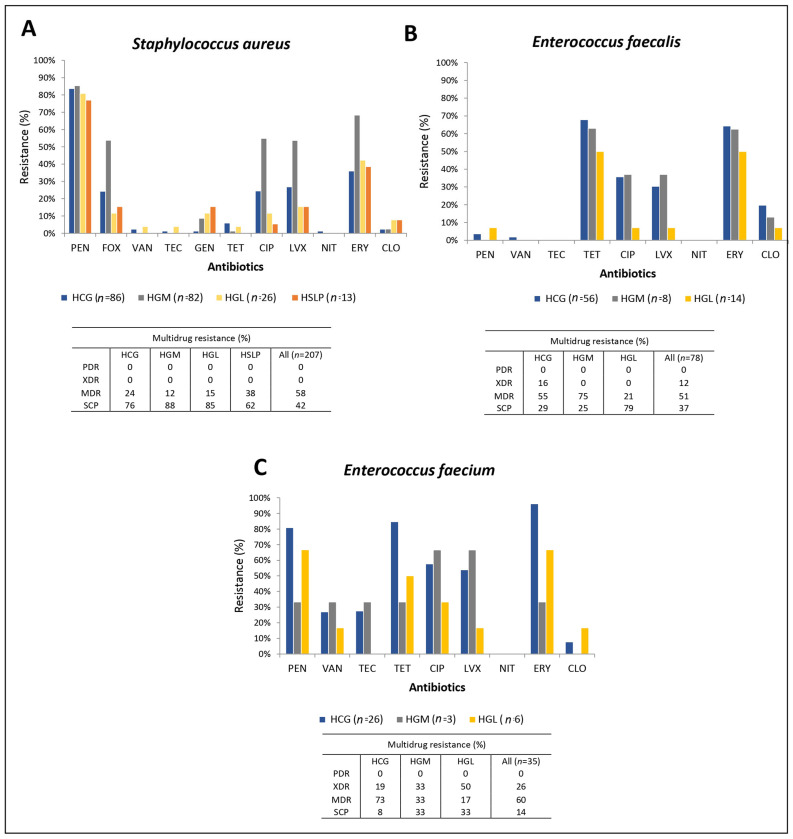
Antimicrobial resistance rates and multidrug resistance in Gram-positive isolates from four hospitals in Mexico. (**A**) *Staphylococcus aureus*, (**B**) *Enterococcus faecalis*, (**C**) *Enterococcus faecium.* PEN: penicillin, FOX: cefoxitin, VAN: vancomycin, TEC: teicoplanin, GEN: gentamicin, TET: tetracycline, CIP: ciprofloxacin, LVX: levofloxacin, NIT: nitrofurantoin, ERY: erythromycin, CLO: chloramphenicol. PDR: pandrug-resistant, XDR: extensively drug-resistant, MDR: multidrug-resistant, SCP: susceptible HCG, Hospital Civil de Guadalajara “Fray Antonio Alcalde” in Guadalajara, Jalisco; HGM, Hospital General de México “Dr. Eduardo Liceaga” in Mexico City; HGL, Hospital General de León in León, Guanajuato; and HSLP, Hospital Central “Dr. Ignacio Morones Prieto” in San Luis Potosí.

The resistance rates of Gram-positive bacteria in the four hospitals are shown in Figure 2. *S. aureus* exhibited resistance rates of up to 83.6% for PEN, 33.8% for FOX (FOX, surrogate for methicillin resistance), and 6.3% for GEN, while remaining almost fully susceptible to VAN and TEC. *E. faecium* showed 74.3% resistance to PEN, 28.6% to VAN, and 27.7% to TEC. In contrast, *E. faecalis* was 96% susceptible to PEN and 99% susceptible to both VAN and TEC. More than half of the *S. aureus*, *E. faecium*, and *E. faecalis* isolates (51–60%) exhibited MDR (Figure 2).

The variability in resistance rates between hospitals revealed that most *A. baumannii* isolates were XDR or PDR across the four hospitals. In contrast, *P. aeruginosa* isolates from HGM exhibited higher resistance rates, with many classified as PDR or MDR, compared to isolates from HCG, HSLP, and HGL. For *K. pneumoniae*, resistance rates were similar across hospitals, with most isolates being MDR.

In *E. cloacae*, isolates from HGM showed higher resistance levels than those from HCG, HGL, and HSLP. Among Gram-positive pathogens, *S. aureus* isolates from HGM presented higher resistance rates than those from the other hospitals, while *E. faecium* showed similar resistance rates across the three hospitals where it was collected. For *E. faecalis*, isolates from HGM and HCG exhibited the highest resistance rates.

Among *K. pneumoniae* isolates, 70% (143/204) were resistant and intermediate to β-lactams. Phenotypic tests confirmed ESBL production in 72.7% (104/143) of these isolates. In addition, 22% (31/139) were resistant to carbapenems, and 100% (31/31) of these carbapenem-resistant isolates were confirmed as carbapenemase producers. *E. cloacae* isolates from HGL and HSLP were β-lactams susceptible (Table 2).

### 3.3. Characterization of Resistance Genes in the ESKAPE Pathogens

The results of resistance to β-lactams and carbapenems and the resistance genes associated with these phenotypes in Gram-negative bacteria from the four hospitals are shown in Table 3. Among Gram-negative bacteria, the *bla*_TEM_ gene was identified in *K. pneumoniae* and *A. baumannii* across the four hospitals, and specifically in *E. cloacae* from HCG and HGM (13%), but was not detected in *P. aeruginosa*. The *bla*_SHV_ gene was exclusively present in *K. pneumoniae* from all hospitals, while the *bla*_CTX-M_ gene was detected in *K. pneumoniae* and *E. cloacae* from HCG and HGM. Regarding carbapenemases, *A. baumannii* isolates showed the highest frequencies, particularly with *bla*_OXA-24_ and *bla*_OXA-23_. In *K. pneumoniae*, 62% of isolates from HCG carried the MBL *bla*_NDM_, and two isolates harbored *bla*_KPC_. By contrast, only a small proportion of *P. aeruginosa* isolates carried *bla*_VIM_.

Phenotypic tests performed on Gram-positive isolates showed that *S. aureus* from HGM exhibited the highest resistance to cefoxitin (53.7%) and a high positivity rate in the cefinase test (97.1%). In the D-test, the highest prevalence of inducible clindamycin resistance was observed in *S. aureus* isolates from HCG (22.6%). Among *Enterococcus* species, the high-level aminoglycoside resistance (HLAR) test was more frequent in *E. faecium* isolates from HCG (38.5%) (Table 4).

The *vanA* gene was consistently detected in vancomycin-resistant nine *E. faecium* and in one *E. faecalis* isolate. The *mecA* gene was identified in 88.6% of *S. aureus* isolates. Aminoglycoside resistance genes *acc(6′)-Ie-aph(2″)-Ia, ant(4′)-Ia, acc(6′-aph(2)*, a*ph(3′)-III*, and were detected in isolates from HCG, HGM, and HGL. Macrolide resistance was associated with *ermA* and *ermC* in *S. aureus*, with *mrsA* additionally detected in *S. aureus* isolates from HGM and HGL. In contrast, *ermB* was more prevalent in enterococci. Finally, the *tetM* gene was predominant among tetracycline-resistant enterococci (Table 5).

Analysis of gene coexistence in Gram-negative bacteria revealed that the most frequent ESBL combination was *bla*_CTX-M_ + *bla*_TEM_ + *bla*_SHV_, detected in 48 *K. pneumoniae* isolates from HCG, followed by *bla*_CTX-M_ + *bla*_SHV_ in 19 isolates of the same species (Table 6). Among combinations including both ESBL and carbapenemase genes, the most frequent was *bla*_OXA-24_ + *bla*_TEM_ in 18 *A. baumannii* isolates from HCG, followed by *bla*_NDM_ + *bla*_CTX-M_ + *bla*_TEM_ + *bla*_SHV_ in 14 *K. pneumoniae* isolates from the same hospital. In Gram-positive bacteria, the most frequent coexistence in *S. aureus* was *blaZ* + *mecA* + *ermA*, identified in 42 isolates from HGM (Table 7). In *E. faecium*, the predominant combination was *blaZ* + *acc(6′)-aph(2)* + *aph(3′)-III* + *acc(6′)-Ie-aph(2″)-Ia* + *tetM*, identified in five isolates from HCG. In *E. faecalis*, the most common coexistence was *acc(6′-aph(2) + aph(3′)-III* + *acc(6′)-Ie-aph(2″)-Ia* + *tetM*, observed in 10 isolates from HCG (Table 8).

### 3.4. Genotyping and Clonal Diversity of ESKAPE Bacteria Across Four Mexican Hospitals

**Figure 3:**
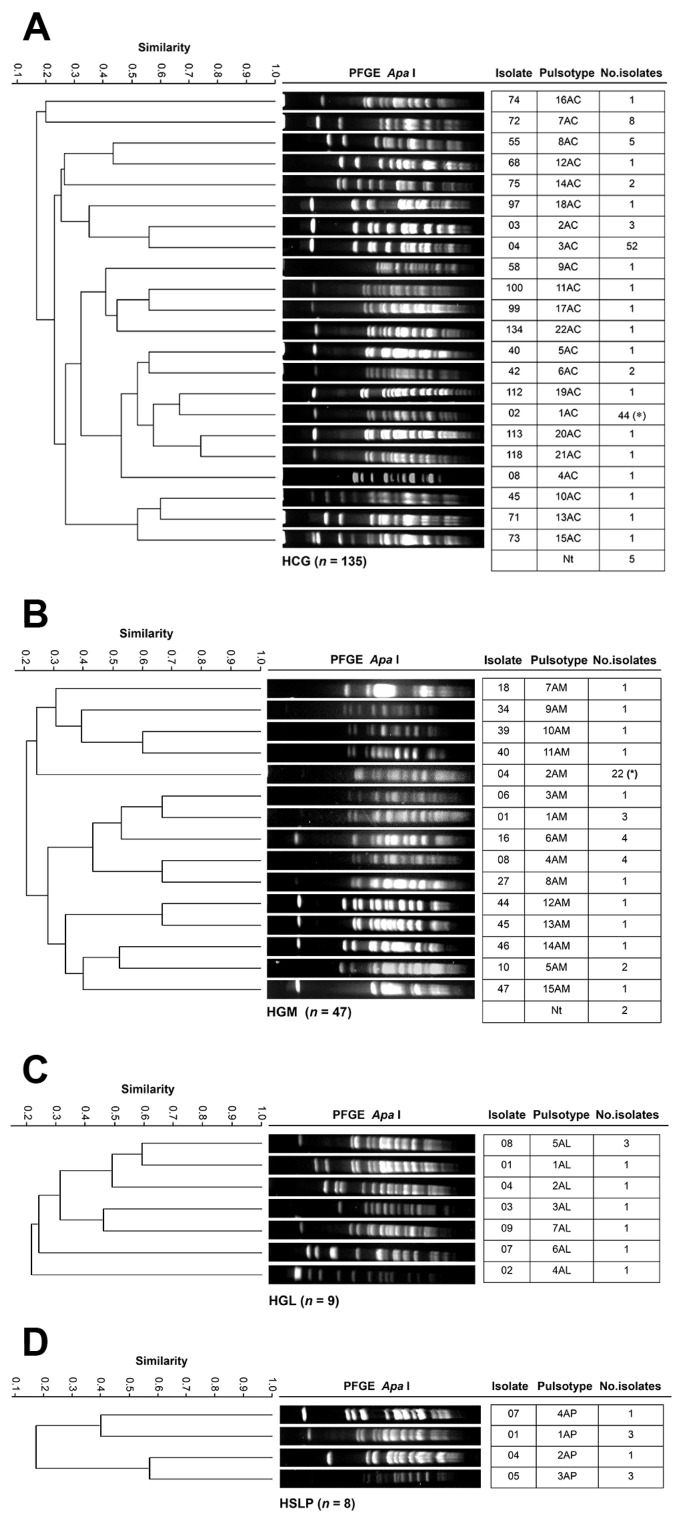
PFGE patterns of *A. baumannii* isolates from tertiary-care hospitals in Mexico. (**A**) Isolates from Hospital Civil de Guadalajara “Fray Antonio Alcalde” (HCG) in Guadalajara, Jalisco, five isolates were non-typeable (Nt); (**B**) Isolates from Hospital General de México “Dr. Eduardo Liceaga” (HGM) in Mexico City, 2 isolates were Nt; (**C**) Isolates from Hospital General de León (HGL) in León, Guanajuato; and (**D**) Hospital Central “Dr. Ignacio Morones Prieto” (HSLP) in San Luis Potosí. The 85% similarity level was used in the cluster designation. PTs in parentheses indicate dissemination across at least two hospitals.

Figure 3 shows the genotyping results of *A. baumannii* isolates across the four hospitals. The 135 HCG isolates collected from 24 hospital departments were grouped into 22 PTs (Figure 3A). The Infectious Diseases Unit accounted for the most considerable number of isolates (63 across nine PTs), with PT 3AC (52 isolates, including 27 from the Infectious Diseases Unit) and PT 1AC (44 isolates, including 26 from the Infectious Diseases Unit) being the most frequent. In Neurosurgery, 14 isolates were identified, distributed across six PTs. At HGM, the 47 isolates from nine departments were classified into 15 PTs (Figure 3B). PT 2AM was the most common, with 22 isolates (11 from the Respiratory Department), which also harbored the most considerable number of isolates overall (18 across six PTs). The nine HGL isolates from six departments were assigned to seven PTs (Figure 3C). PT 5AL was identified in Internal Medicine (2/3) and in the Emergency Department (1/3) during different months (August, October, and November), reflecting high clonal variability. The eight HSLP isolates from five departments were grouped into four PTs (Figure 3D). PT 1AP was present in Internal Medicine and the Intensive Care Unit, while PT 3AP was detected in Surgery. Overall, clonal dissemination of *A. baumannii* was observed: HCG PT 1AC(*) was also identified in HGM, corresponding to PT 2AM(*). However, no PT associated with a nosocomial outbreak was detected in any of the hospitals.

**Figure 4:**
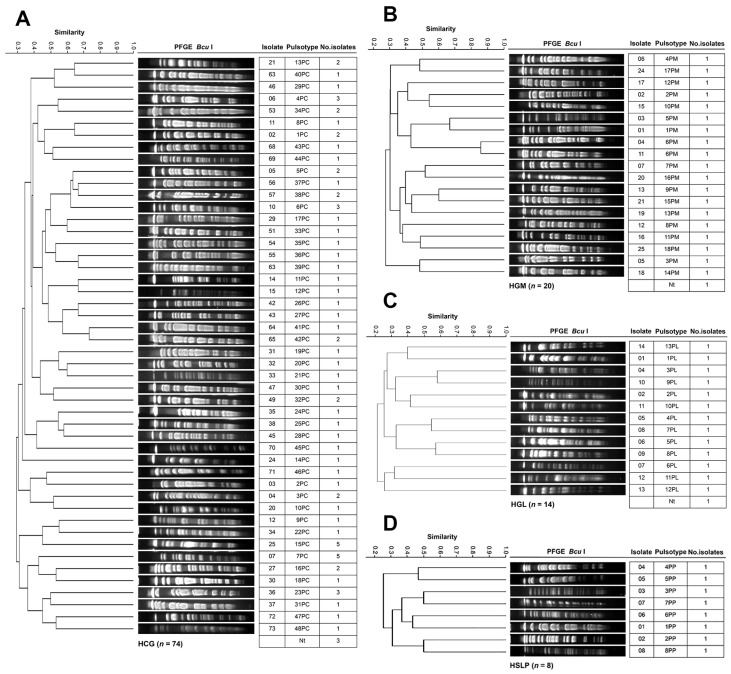
PFGE patterns of *P. aeruginosa* isolates from tertiary-care hospitals in Mexico. (**A**) Isolates from Hospital Civil de Guadalajara “Fray Antonio Alcalde” (HCG) in Guadalajara, Jalisco, three isolates were non-typeable (Nt); (**B**) Isolates from Hospital General de México “Dr. Eduardo Liceaga” (HGM) in Mexico City, one isolate was Nt; (**C**) Isolates from Hospital General de León (HGL) in León, Guanajuato, one isolate was Nt; and (**D**) Hospital Central “Dr. Ignacio Morones Prieto” (HSLP) in San Luis Potosí. The 85% similarity level was used in the cluster designation.

Figure 4 shows the genotyping results of *P. aeruginosa* across the four hospitals. The 74 HCG isolates from 23 departments were grouped into 48 PTs (Figure 4A). The Infectious Diseases Unit accounted for the most considerable number of isolates (19 across 18 PTs), followed by Nephrology (7 across 5 PTs), Pediatrics (7 across 7 PTs), and Internal Medicine (5 across 5 PTs). PTs 7PC and 15PC were the most frequent, with five isolates each. At HGM, the 20 isolates from 11 departments were classified into 18 PTs (Figure 4B). PT 6PM was identified in the Emergency Department in April and in the Infectious Diseases Unit in June. The 14 HGL isolates from 11 departments were assigned to 13 PTs (Figure 4C). Internal Medicine accounted for the highest number of isolates (four across four PTs), followed by the Pediatric Emergency Department (three across two PTs). At HSLP, the eight isolates from six departments were classified into 8 PTs (Figure 4D). The Pediatric Department had the largest number of isolates (three across three PTs). No PT associated with clonal dissemination or nosocomial outbreak was detected in any of the hospitals.

**Figure 5:**
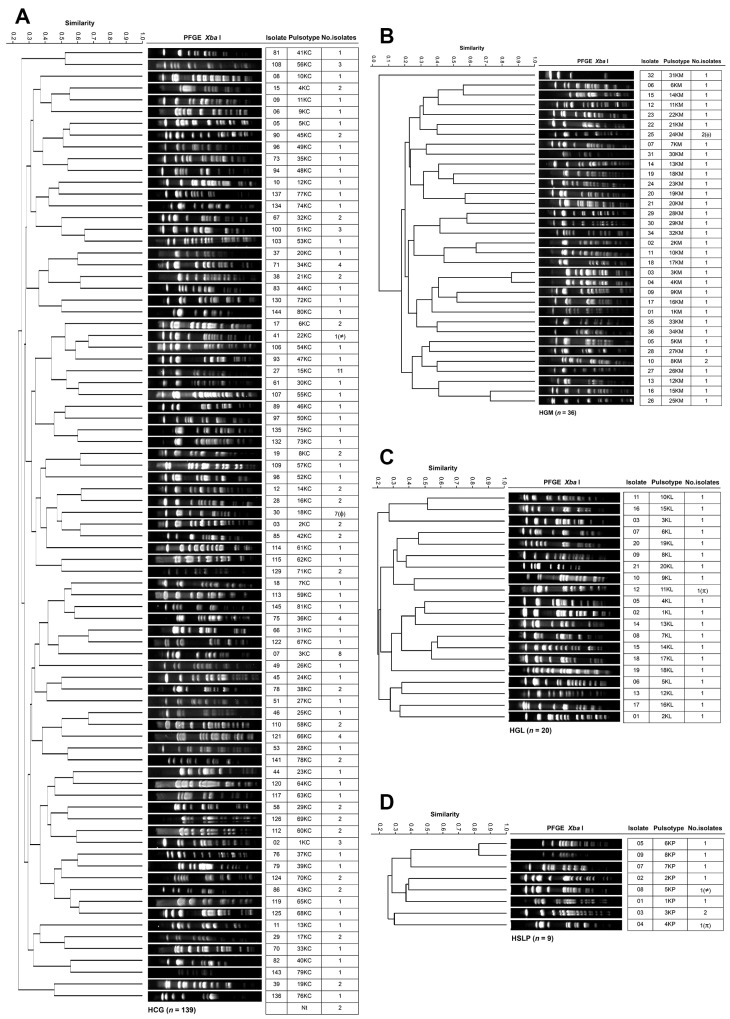
PFGE patterns of *K. pneumoniae* isolates from tertiary-care hospitals in Mexico. (**A**) Isolates from Hospital Civil de Guadalajara “Fray Antonio Alcalde” (HCG) in Guadalajara, Jalisco, two isolates were non-typeable (Nt); (**B**) Isolates from Hospital General de México “Dr. Eduardo Liceaga” (HGM) in Mexico City; (**C**) Isolates from Hospital General de León (HGL) in León, Guanajuato, one isolate was Nt; and (**D**) Hospital Central “Dr. Ignacio Morones Prieto” (HSLP) in San Luis Potosí. The 85% similarity level was used in the cluster designation. PTs in parentheses indicate dissemination across at least two hospitals.

Figure 5 shows the genotyping results of *K. pneumoniae* across the four hospitals. The 139 HCG isolates from 27 departments were grouped into 81 PTs (Figure 5A). The Infectious Diseases Unit accounted for the most considerable number of isolates (36 across 28 PTs), followed by Neurosurgery (16 across 14 PTs), Pediatrics (13 across 11 PTs), and Internal Medicine (10 across 9 PTs). PTs 15KC, 3KC, and 18KC were the most frequent, with 11, 8, and 7 isolates, respectively. At HGM, the 36 isolates from 11 departments were distributed into 34 PTs (Figure 5B). PTs 8KM and 24KM were the most common, detected in the Respiratory Department (seven across six PTs) and the Intensive Care Unit (six across six PTs). The 20 HGL isolates from six departments were assigned to 20 PTs (Figure 5C). The Pediatric Oncology Department (five across five PTs), Internal Medicine (five across five PTs), and the Intensive Care Unit (four across four PTs) accounted for the most significant number of isolates. At HSLP, the nine isolates from six departments were classified into eight PTs (Figure 5D). PT 3KP was identified in Surgery (two isolates in April). In *K. pneumoniae*, evidence of clonal dissemination was observed: PT 22KC(≠) from HCG was also found in HSLP, corresponding to PT 5KP(≠); PT 18KC(ϕ) from HCG was detected in HGM, corresponding to PT 24KM(ϕ); and PT 11KL(π) from HGL was identified in HSLP, corresponding to PT 4KP(π). However, no PT associated with a nosocomial outbreak was identified in any of the hospitals. The analysis of antimicrobial resistance profiles and resistance gene carriage among isolates belonging to the same PT that disseminated across different hospitals revealed heterogeneity between them. Nevertheless, most isolates harbored BLEEs, whereas only a few members of PT 18KC(ϕ) carried the NDM carbapenemase gene.

**Figure 6:**
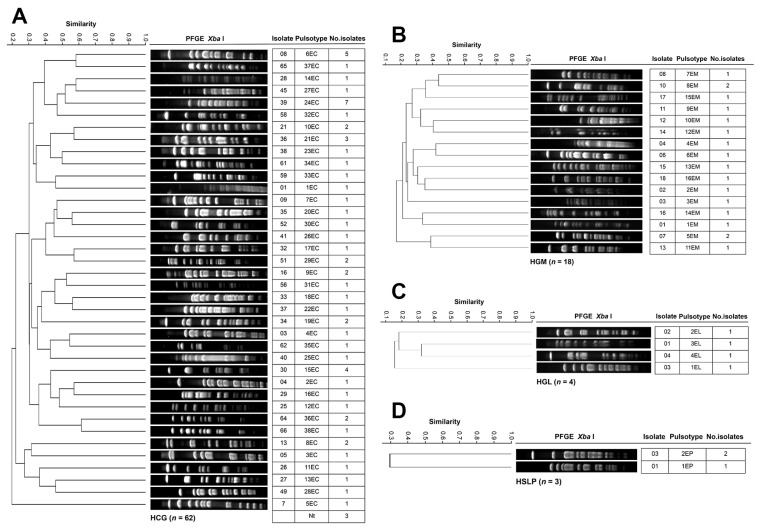
PFGE patterns of *E. cloacae* isolates from tertiary-care hospitals in Mexico. (**A**) Isolates from Hospital Civil de Guadalajara “Fray Antonio Alcalde” (HCG) in Guadalajara, Jalisco, three isolates were non-typeable (Nt); (**B**) Isolates from Hospital General de México “Dr. Eduardo Liceaga” (HGM) in Mexico City; (**C**) Isolates from Hospital General de León (HGL) in León, Guanajuato, one isolate was Nt; and (**D**) Hospital Central “Dr. Ignacio Morones Prieto” (HSLP) in San Luis Potosí. The 85% similarity level was used in the cluster designation.

Figure 6 shows the genotyping results of *E. cloacae* isolates across the four hospitals. The 62 HCG isolates from 19 departments were grouped into 38 PTs (Figure 6A). Nephrology accounted for the most considerable number of isolates (13 across nine PTs), followed by the Infectious Diseases Unit (7 across six PTs), Neurosurgery (7 across five PTs), and Pediatrics (6 across six PTs). PTs 15EC and 36EC were detected in Nephrology with four and two isolates, respectively. PTs 24EC, 6EC, and 21EC were the most frequent overall, with seven, five, and three isolates, respectively. At HGM, 18 isolates from 10 departments were assigned to 16 PTs (Figure 6B). PTs 5EM 2/2 and 8EM, each with two isolates, were detected in Hematology (May and June), and in the Respiratory Department and Nephrology, respectively. The four HGL isolates from three departments were distributed among four PTs (Figure 6C), with two of them detected in the Emergency Department. The three HSLP isolates from Pediatrics were classified into two PTs (Figure 6D). No PT associated with clonal dissemination or nosocomial outbreak was identified in any of the hospitals.

**Figure 7:**
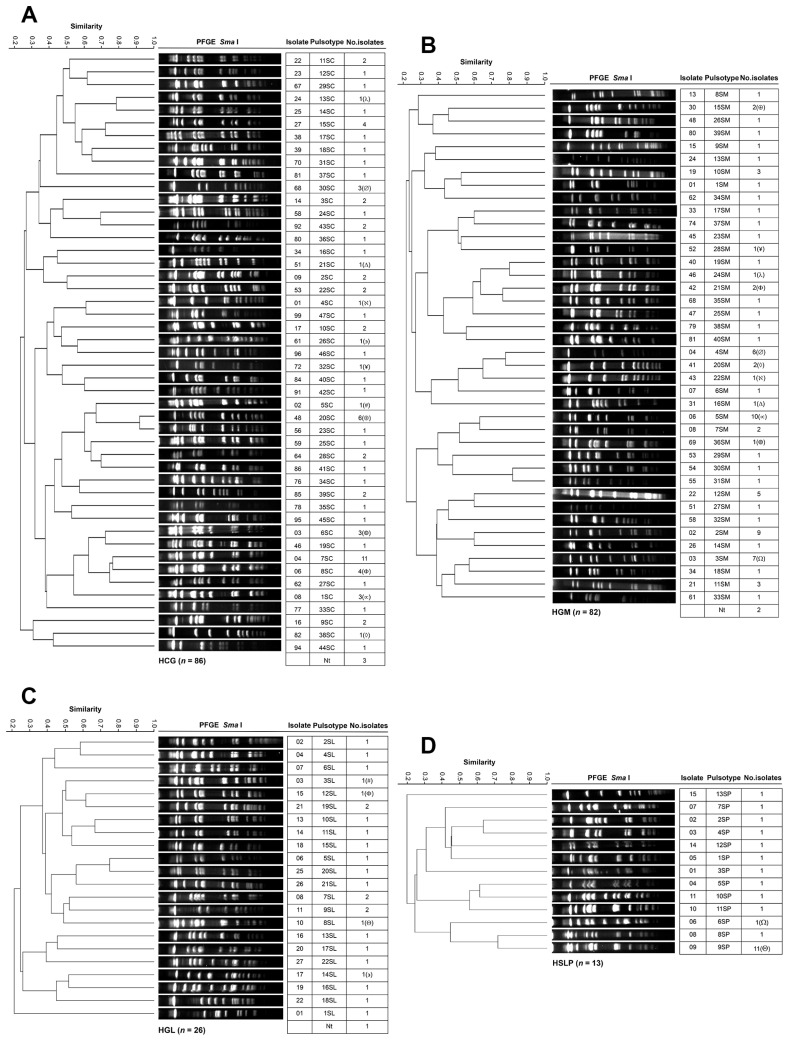
PFGE patterns of *S. aureus* isolates from tertiary-care hospitals in Mexico. (**A**) Isolates from Hospital Civil de Guadalajara “Fray Antonio Alcalde” (HCG) in Guadalajara, Jalisco, three isolates were non-typeable (Nt); (**B**) Isolates from Hospital General de México “Dr. Eduardo Liceaga” (HGM) in Mexico City, two isolate was Nt; (**C**) Isolates from Hospital General de León (HGL) in León, Guanajuato, one isolate was Nt; and (**D**) Hospital Central “Dr. Ignacio Morones Prieto” (HSLP) in San Luis Potosí. The 85% similarity level was used in the cluster designation. PTs in parentheses indicate dissemination across at least two hospitals.

Figure 7 shows the genotyping results of *S. aureus* isolates across the four hospitals. The 86 HCG isolates from 18 departments were grouped into 47 PTs (Figure 7A). Nephrology harbored the largest number of isolates (22 across 17 PTs), followed by the Infectious Diseases Unit (11 across 11 PTs), Internal Medicine (8 across 8 PTs), Gastroenterology (6 across 4 PTs), and Geriatrics (6 across 5 PTs). PT 7SC *(n* = 11) was widely distributed across seven departments, including three isolates in Neurosurgery, two in Geriatrics, and two in Transplants. PT 20SC was identified in four departments (In Nephrology and Gastroenterology, with two isolates in each). The 82 HGM isolates from 20 hospital departments were classified into 40 PTs (Figure 7B). PT 5SM was the most predominant with 10 isolates, followed by PTs 2SM, 3SM, 4SM, and 12SM, with 9, 7, 6, and 5 isolates, respectively. The departments with the highest number of isolates were the Respiratory Department (15 across 10 PTs), Internal Medicine (14 across 11 PTs), Emergency Department (12 across 10 PTs), Intensive Care Unit (7 across 7 PTs), Urology (6 across 6 PTs), and Neurology (6 across 4 PTs). The 26 HGL isolates from six departments were assigned to 22 PTs (Figure 7C). Internal Medicine harbored the most significant number of isolates (eight across eight PTs), followed by the Emergency department (eight across five PTs), two of which belonged to 7SL. PTs 9SL and 19SL, with two isolates each, were distributed across different departments. The 13 HSLP isolates from five departments were classified into 13 PTs (Figure 7D). Internal Medicine accounted for the most significant number of isolates (nine across nine PTs).

In *S. aureus*, clonal dissemination was observed: PT 13SC(λ) from HCG was identified in HGM, corresponding to PT 24SM(λ); PT 30SC(∅) from HCG was detected in HGM, corresponding to PT 4SM(∅); PT 21SC(Δ) from HCG was identified in HGM, corresponding to PT 16SM(Δ); PT 4SC(ℵ) from HCG was detected in HGM, corresponding to PT 22SM(ℵ); PT 26SC(∍) from HCG was identified in HGL, corresponding to PT 14SL(∍); PT 32SC(¥) from HCG was detected in HGM, corresponding to PT 28SM(¥); PT 5SC(#) from HCG was identified in HGL, corresponding to 3SL(#); PT 20SC(⊕) from HCG was detected in HGM, corresponding to PT 15SM(⊕); PT 6SC(©) from HCG was identified in HGM, corresponding to PT 36SM(©); PT 8SC(Φ) from HCG was detected in HGL, corresponding to PT 12SL(Φ) and in HGM, corresponding to PT 21SM(Φ); PT 1SC(∝) from HCG was identified in HGM, corresponding to PT 5SM(∝); PT 38SC(◊) from HCG was detected in HGM, corresponding to PT 20SM(◊); PT 3SM(Ω) from HGM was identified in HSLP, corresponding to PT 6SP(Ω); and PT 8SL(Θ) from HGL was detected in HSLP, corresponding to PT 9SP(Θ). No PT associated with a nosocomial outbreak was identified in any of the hospitals.

**Figure 8:**
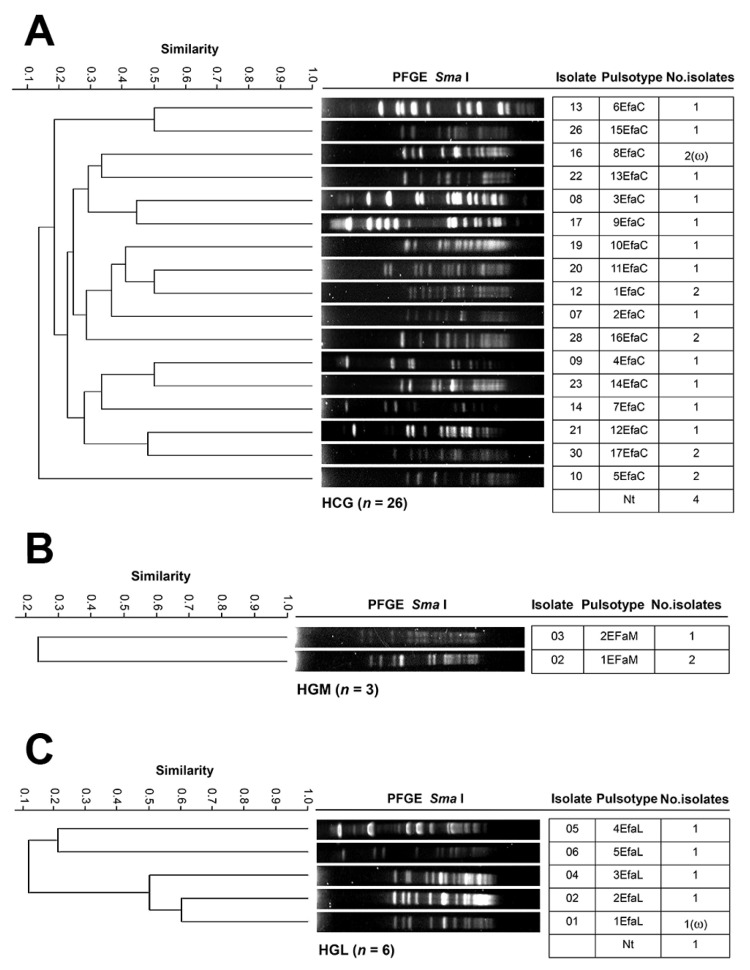
PFGE patterns of *E. faecium* isolates from tertiary-care hospitals in Mexico. (**A**) Isolates from Hospital Civil de Guadalajara “Fray Antonio Alcalde” (HCG) in Guadalajara, Jalisco, four isolates were non-typeable (Nt); (**B**) Isolates from Hospital General de México “Dr. Eduardo Liceaga” (HGM) in Mexico City; and (**C**) Isolates from Hospital General de León (HGL) in León, Guanajuato, one isolate was Nt; the 85% similarity level was used in the cluster designation. PTs in parentheses indicate dissemination across at least two hospitals.

Figure 8 shows the genotyping results of *E. faecium* across the four hospitals. The 26 HCG isolates from 10 departments were grouped into 17 PTs (Figure 8A). The Infectious Diseases Unit accounted for the most considerable number of isolates (nine across seven PTs), followed by Pediatrics (three across three PTs). PTs 1EFaC, 5EFaC, 8EFaC, 16EFaC, and 17EFaC were the most frequent, with two isolates each. The three HGM Efa isolates from two departments were classified into two PTs (Figure 8B); PT 2EFaM was detected in the Surgery and in the Intensive Care unit. The six HGL isolates from three departments were assigned to five PTs in five different departments (Figure 8C). PT 8EfaC(ω) was detected in HCG, corresponding to PT 1EfaL(ω) from HGL. No *E. faecium* isolates were identified in samples from HSLP, indicating the absence of this species in that hospital during the study period. No PT associated with clonal dissemination or nosocomial outbreak was identified in any of the hospitals.

**Figure 9:**
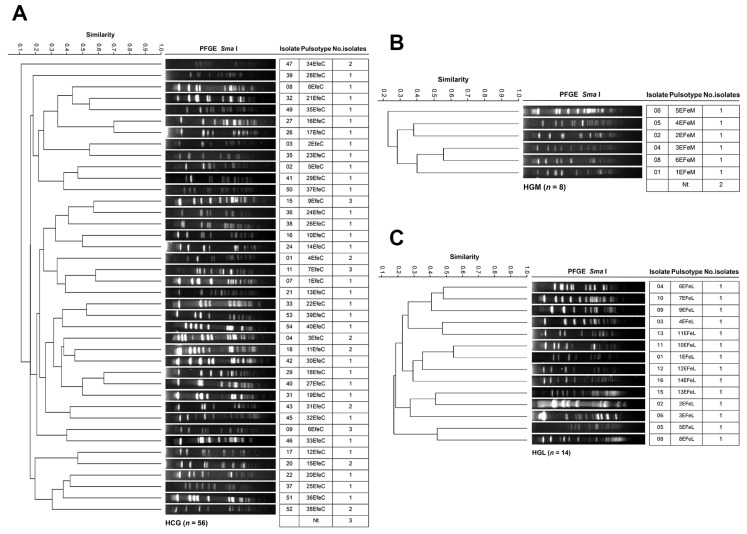
PFGE patterns of *E. faecalis* isolates from tertiary-care hospitals in Mexico. (**A**) Isolates from Hospital Civil de Guadalajara “Fray Antonio Alcalde” (HCG) in Guadalajara, Jalisco, three isolates were non-typeable (Nt); (**B**) Isolates from Hospital General de México “Dr. Eduardo Liceaga” (HGM) in Mexico City, two isolates were Nt; and (**C**) Isolates from Hospital General de León (HGL) in León, Guanajuato; the 85% similarity level was used in the cluster designation.

Figure 9 shows the results of *E. faecalis* genotyping across the four hospitals. The 56 HCG isolates from 17 departments were grouped into 40 PTs (Figure 9A). The Infectious Diseases Unit harbored the largest number of isolates (15 across 13 PTs), followed by the Intensive Care Unit (7 across 6 PTs), and Pediatrics (6 across 6 PTs). PTs 6EFeC, 7EFeC, and 9EFeC were the most frequent, each with three isolates distributed across different departments. The eight HGM isolates from three departments were classified into six PTs (Figure 9B). Internal Medicine accounted for the largest number of isolates (three in three PTs), followed by the Emergency Department (two across two PTs). The 14 HGL isolates from six departments were assigned to 14 PTs (Figure 9C). The Intensive Care Unit had the highest number of isolates (five across five PTs), followed by Internal Medicine (three across three PTs). No *E. faecium* isolates were identified in samples from HSLP, indicating the absence of this species in that hospital during the study period. No PT associated with clonal dissemination or nosocomial outbreak was identified in any of the hospitals.

## 4. Discussion

This multicenter study provides a comprehensive overview of the burden of nosocomial bacteremia caused by ESKAPE pathogens in Mexican hospitals. The differences in bacterial distribution reflect the number of patients attended in each hospital (most isolates originated from the HCG [62.7%], followed by the HGM [22.9%], the HGL [10.1%], and the HSLP [4.3%]), the characteristics of the patient populations they serve, and their geographic location within Mexico. The considerable number of isolates analyzed indicates an elevated level of antimicrobial resistance across the hospitals, which may inform the implementation of enhanced infection control strategies and evidence-based empirical treatment guidelines.

The prevalence of ESKAPE pathogens showed that *S. aureus* was the most frequent bacterium, followed by *K. pneumoniae*, *A. baumannii*, *P. aeruginosa*, *E. cloacae*, *E. faecalis*, and *E. faecium,* a pattern aligned with those reported by the National Epidemiological Surveillance Network (PUCRA) [33] and the Resistance Research and Surveillance Network (INVIFAR) [34]. However, unlike those findings, *A. baumannii* ranked among the top three, probably because HGM and HCG served as referral centers for critically ill COVID-19 patients [35]. Previous studies noted that coinfection with *A. baumannii* in severe SARS-CoV-2 pneumonia is between 1% and 28% [36], which could even have led to bacteremia. These data support the epidemiological significance of *A. baumannii* in our study hospitals during the COVID-19 pandemic.

It is possible that *A. baumannii* may have improved levels of antimicrobial resistance in hospitals during the COVID-19 pandemic. According to Golli et al., many critically ill COVID-19 patients were treated with empirical antibiotics for suspected secondary bacterial infections without microbiological confirmation, thus increasing antibiotic exposure unnecessarily [37]. Empirical antibiotics, including broad-spectrum antibiotics such as β-lactams, macrolides, or fluoroquinolones, were potentially being used in patients without bacterial coinfection and could possibly create selective pressure on a variety of bacterial species, contributing to MDR organisms and bloodstream infections. Overall, there needs to be enhancements in antimicrobial stewardship and healthcare providers' adherence to infection prevention practices in the hospital setting in the post-COVID-19 pandemic period.

Among Gram-negative bacteria, *K. pneumoniae* showed lower cephalosporin resistance to was lower than previously documented in Mexican hospitals [34,38]. Nevertheless, the high proportion of potential ESBL producers (79%) indicates that β-lactamase-mediated resistance continues to diversify and limit therapeutic options, while there was an apparent decrease in overall resistance rates. In this collection, CTX-M-type β-lactamases were the most common, followed closely by members of the SHV and TEM families, which is consistent with global patterns [39]. Carbapenem resistance (22%) was similar to findings from national surveillance [14] and consisted of both *bla*_NDM-1_ and *bla*_KPC_, as previously reported in Mexico [40]. Colistin resistance (6.8%) surpassed levels reported earlier at the national level [41]. The frequent co-occurrence of *bla*_NDM_ among colistin-resistant isolates suggests that combined carbapenem and polymyxin resistance is emerging through persistence of high-risk clones rather than sporadic acquisition events [41]. In *E. cloacae*, β-lactams showed the highest resistance rates, and carbapenem resistance (20.8%) was slightly higher than that reported three years earlier in Brazil [42], emphasizing the need for continued molecular monitoring to detect early dissemination of carbapenemase-producing *E. cloacae.*

Previous studies from our group showed similarly elevated levels of resistant *A. baumannii* from HCG and HGM [12,35], and this trend continues as PDR and XDR *A. baumannii* remain prevalent. The occurrence of OXA-type carbapenemase (*bla*_OXA-24_ and *bla*_OXA-23_) was similar to global data [43], suggesting that local persistence stems largely from clonal expansion of OXA-producing strains. Although polymyxin continues to be effective against the majority of isolates, the data must be interpreted with caution, as emerging polymyxin resistance is frequently reported worldwide [44]. For *P. aeruginosa*, the overall low rate of ceftazidime and cefepime resistance indicates that there may still be reasonable options for β-lactams when used in conjunction with susceptibility testing. Levels of MDR were comparable to national data [40], suggesting that *P. aeruginosa* resistance has remained stable. However, the predominance of the *bla*_VIM_ gene among carbapenem-resistant isolates reflects the ongoing dissemination of metallo-β-lactamase-producing clones that complicate infection management in intensive care settings.

Among Gram-positive bacteria, *S. aureus* exhibited a high prevalence of methicillin-resistant isolates (MRSA), underscoring its persistent burden in hospital environments. The predominance of the *mecA* gene, possible *mecC* variants [45], indicates genetic diversity underlying methicillin resistance and emphasizes the importance of molecular surveillance to detect emerging *mec* gene subtypes. Aminoglycoside resistance, mainly mediated by *aac(6′)-Ie-aph(2″*)-*Ia*, was consistent with reports from Iran [46], while erythromycin resistance was primarily associated with *ermA*, as reported in Latin American [47]. The frequent presence of these three genes (*blaZ*, *mecA*, and *ermA*) in the same background also supports that MDR *S. aureus* originated through the accumulation of determinants of resistance. Resistance to fluoroquinolone was at levels similar to those reported by INVIFAR [14] and indicate stability over time.

The vancomycin resistance rates remain high in *E. faecium* and are similar to other reports in hospitals in Mexico [8], but lower than rates reported in the United States and Europe; *E. faecalis* had lower resistance rates consistent with trends reported globally[48]. The presence of the *vanA* gene in all vancomycin-resistant isolates supports its position as the main glycopeptide resistance determinant, in agreement with previous studies from multiple regions of Iran [49]. The observed aminoglycoside resistance, mainly mediated by *aph(3′)-IIIa* and *aac(6′)-Ie-aph(2″)*, correspond with studies conducted in Asia and the Americas [49,50]. This supports the suggestion that MDR in enterococci can develop via the accumulation of different resistance genes within the same genomic context and that a framework for horizontal gene exchange and persistence under antibiotic pressure may occur. Moreover, elevated resistance to tetracyclines and fluoroquinolones, particularly among *E. faecium*, further underscores this species’ capacity for adaptation to antimicrobial pressure.

This study has some limitations, including the absence of clinical data (e.g., comorbidities, prior antibiotic exposure, length of hospitalization, and mortality). The limitations, outlined above, associated with hospital overcrowding during the rise of the COVID-19 pandemic, affect the interpretation of the clinical effect of AMR. Additionally, with temporal trend analysis not performed, we cannot gauge if resistance emergence has been seasonally affected. Nevertheless, clonal analysis provided a key contribution to epidemiological research by documenting the emergence and persistence of resistant clones and their transmissibility between hospitals.

PFGE continued to be the gold standard for ascertaining resultant clonal relatedness, demonstrating the emergence of specific PTs, persistently comprising multiple healthcare-associated outbreaks within the hospital, which indicated long-term endemicity [11]. For instance, *A. baumannii* PT 1AC from HCG (44 isolates, mostly XDR) was distributed across 13 departments—mainly the Infectious Diseases Unit (59%)—and corresponds to a clone first identified at HCG in 2007, previously linked to a nosocomial [21]. Similarly, PT 2AM from HGM (22 isolates), associated with a COVID-19 outbreak [35], and PT 3AC (52 isolates) from multiple hospital areas, also showed XDR profiles. Other clones included *K. pneumoniae* PT 15KC (11 isolates; 9 XDR and 3 MDR), and PT 18KC (6 XDR isolates), as well as *E. cloacae* PT 6EC and *P. aeruginosa* PTs 7PC and 15PC (5 isolates each). Despite their wide distribution, no active nosocomial outbreaks were confirmed by hospital infection control units. These findings demonstrate that certain *A. baumannii* lineages possess exceptional environmental persistence [51], posing significant containment challenges.

In contrast, *P. aeruginosa* displayed high levels of clonal diversity across hospitals, and *S. aureus* showed substantial interhospital dissemination involving 14 different PTs. The other notable findings were the *K. pneumoniae f*ound with three PTs, an *E. faecalis* with one PT, and an *A. baumannii* with one PT, with interhospital dissemination. Similar multicenter transmission has been reported for *S. aureus* and *K. pneumoniae* [10]. The widespread dissemination of *S. aureus* PTs provides evidence of the organism’s high transmissibility and resilience in the environment. Given the structural characteristics of the Mexican healthcare system, local patient transfer networks could facilitate regional and interstate transmission and emphasize the need for long-term genomic surveillance to identify and break transmission at the local and national levels.

## 5. Conclusions

In summary, this multicenter study reveals the substantial burden and complex epidemiology of ESKAPE pathogens causing nosocomial bacteremia in Mexican tertiary-care hospitals. The persistence of MDR and XDR strains, particularly among *A. baumannii* and *K. pneumoniae*-along with the detection of high-risk clones and interhospital dissemination of *S. aureus* and *K. pneumoniae*, emphasizes the dynamic and adaptive nature of these pathogens.

Our results reinforce the urgent need for coordinated infection control programs, ongoing genomic surveillance, and optimized empirical therapy to mitigate the spread of resistance and improve patient outcomes. These efforts are especially critical in the post-COVID-19 era, as healthcare systems continue to face the compounded challenges of antimicrobial resistance and hospital-acquired infections.

## Figures and Tables

**Table 1:** Primers used for PCR amplification of resistance genes in bacteria of the ESKAPE group.

Antimicrobial Resistance	Gene	Sequence 5′–3′	Amplicon Length (bp)	Annealing Temperature (°C)	Reference
Primer sequences for Gram-negative bacteria					
Class A β-lactamase	*bla*_TEM_	F: ATGAGTATTCAACATTTTCG R: TTACCAATGCTTAATCAGTGAG	861	55	[19]
	*bla*_CTX−M−type_	F: CGCTTTGCGATGTGCAG R: ACCGCGATATCGTTGGT	550	52	[20]
	*bla*_SHV_	F: ATGCGTTATATTCGCCTGTGTATT R: TTAGCGTTGCCAGTGCTCGATC	861	50	[12]
Class A Carbapenemase	*bla*_KPC_	F: TCACTGTATCGCCGTCTAGTTCTG R: TTACTGCCCGTTGACGCCCAATC	875	58	[12]
Class B β-lactamase	*bla*_VIM_	F: GAGTGGTGAGTATCCGACAGTCAACGAAAT R: AGAGTCCTTCTAGAGAATGCGTGGGAATCT	389	58	[12]
	*bla*_NDM_	F: GTCTGGCAGCACACTTCCTATCTC R: GTAGTGCTCAGTGTCGGCATCACC	516	58	[12]
	*bla*_IMP_	F: GCATTGCTACCGCAGCAGAGTCTTTG R: GCTCTAATGTAAGTTTCAAGAGTGATGC	647	58	[12]
Class D β-lactamase	*bla*_OXA−40−like_	F: TCTAGTTTCTCTCAGTGCATGTTCATC R: CATTACGAATAGAACCAGACATTCC	749	58	[12]
	*bla*_OXA−51−like_	F: ATGAACATTMAARCRCTCTTACTTA R: CTATAAAATACCTAATTMTTCTAA	825	50	[21]
	*bla*_OXA-23-like_	F: ATATTTTACTTGCTATGTGGTTGCTTC R: ATAATTCATTACGTATAGATGCCGGCA	752	55	This study
Primer sequences for Gram-positive bacteria					
β-lactam resistance	*mecA*	F: TGGCTATCGTGTCACAATCG R: CTGGAACTTGTTGAGCAGAG	310	52	[22]
	*blaZ*	F: AACACCTGCTGCTTTC R: CTCTTGGCGGTTTCAC	312	49	[23]
Macrolides	*ermA*	F: ATCGGATCAGGAAAAGGACA R: CACGATATTCACGGTTTACCC	486	52	[24]
	*ermB*	F: AAGGGCATTTAACGACGAAA R: CTGTGGTATGGCGGGTAAGT	423	50	[24]
	*erm*C	F: TGAAATCGGCTCAGGAAAAG R: CAAACCCGTATTCCACGATT	272	52	[24]
	*msr*A	F: TGGTACTGGCAAAACCACAT R: AAACGTCACGCATGTCTTCA	1000	52	[24]
Aminoglycoside	*aac(6′)-Ie-aph(2* *″* *)-Ia*	F: CAGAGCCTTGGGAAGATGAAG R: CCTCGTGTAATTCATGTTCTGGC	348	55	[25]
	*aph(3)-IIIa*	F: GGCTAAAATGAGAATATCACCGG R: CTTTAAAAAATCATACAGCTCGCG	523	55	[25]
	*ant(4)-Ia*	F: CAAACTGCTAAATCGGTAGAAGCC R: GGAAAGTTGACCAGACATTACGAACT	294	55	[25]
	*acc(6′)-aph(2)*	F: CCAAGAGCAATAAGGGCATA R: CACTATCATAACCACTACCG	220	50	[26]
	*aph(3′)-III*	F: GCCGATGTGGATTGCGAAAA R: GCTTGATCCCCAGTAAGTCA	292	50	[23]
	*aph(2* *″* *)-Ic*	F: GAAGTGATGGAAATCCCTTCGTG R: GCTCTAACCCTTCAGAAATCCAGTC	627	55	[27]
Vancomycin	*vanA*	F: GCTATTCAGCTGTACTC R: CAGCGGCCATCATACGG	783	54	[23]
Tetracycline	*tetM*	F: GTGTGACGAACTTTACCGAA R: GCTTTGTATCTCCAAGAACAC	501	55	[28]

**Table 2:** Resistance phenotypes ESBL and carbapenemase production in Gram-negative ESKAPE isolates from four hospitals in Mexico.

Microorganism	*Klebsiella pneumoniae*					*Enterobacter cloacae*			*Acinetobacter baumannii*					*Pseudomonas aeruginosa*				
Hospital (*n*)	HCG (139)	HGM (36)	HGL (20)	HSLP (9)	All (204)	HCG (62)	HGM (18)	All (80)	HCG (135)	HGM (47)	HGL (9)	HSLP (8)	All (199)	HCG (74)	HGM (20)	HGL (14)	HSLP (8)	All (116)
β-lactam-intermediate and resistant isolates (*n*, %)	97, 69.8	29, 80.5	10, 50	9, 100	143, 70	16, 25.8	7, 38.9	23, 28.8	134, 99.6	47, 100	8, 88.9	7, 87.5	196, 98.5	14, 18.9	11, 55	3, 21.4	2, 25	30, 25.9
ESBL phenotype (%)	67.0	96.5	40	100	72.7	N/A	N/A	N/A	N/A	N/A	N/A	N/A	N/A	N/A	N/A	N/A	N/A	N/A
Carbapenem-intermediate and resistant isolates (*n*, %)	31, 22.3	0, 0	0,0	0,0	31, 15.2	7, 11.3	9, 50	16, 20	132, 98	46, 97	6, 66.6	7, 87.5	196, 98.5	36, 48.6	17, 85	14, 100	2, 25	69, 59.5
mCIM phenotype (%)	100	0	0	0	100	0	0	0	N/A	N/A	N/A	N/A	N/A	N/A	N/A	N/A	N/A	N/A

HCG, Hospital Civil de Guadalajara “Fray Antonio Alcalde” in Guadalajara, Jalisco; HGM, Hospital General de México “Dr. Eduardo Liceaga” in Mexico City; HGL, Hospital General de León in León, Guanajuato; and HSLP, Hospital Central “Dr. Ignacio Morones Prieto” in San Luis Potosí. mCIM: modified carbapenem inactivation method. N/A Not applicable.

**Table 3:** Resistance ESBL and carbapenemase genes in Gram-negative ESKAPE isolates from four hospitals in Mexico.

Microorganism		*Klebsiella pneumoniae*					*Enterobacter cloacae*			*Acinetobacter baumannii*					*Pseudomonas aeruginosa*				
Hospital		HCG	HGM	HGL	HSLP	All	HCG	HGM	All	HCG	HGM	HGL	HSLP	All	HCG	HGM	HGL	HSLP	All
ESBL genes (*n*, %)	*bla*_TEM_	64, 66	17, 58.6	3, 30	6, 66.7	90, 62.9	2, 12.5	1, 14.3	3, 13	42, 31	11, 23.4	4, 50	2, 28.6	59, 30.1	0	0	0	0	0
	*bla*_SHV_	91, 93.8	24, 82.8	6, 60	7, 77.8	128, 89.5	0	0	0	0	0	0	0	0	0	0	0	0	0
	*bla*_CTX-M_	89, 91.8	25, 86.2	0	0	114, 79.7	2, 12.5	4, 57.1	6, 26	0	0	0	0	0	0	0	0	0	0
Carbapenemase genes (*n*, %)	*bla*_OXA-24_	0	0	0	0	0	0	0	0	72, 54	1, 2	1, 17	1, 14.3	75, 38.3	0	0	0	0	0
	*bla*_OXA-23_	0	0	0	0	0	0	0	0	15, 11	19, 41	0	0	34, 17.3	0	0	0	0	0
	*bla*_NDM_	21, 67.7	0	0	0	21, 67.7	0	0	0	0	0	0	0	0	0	0	0	0	0
	*bla*_VIM_	0	0	0	0	0	0	0	0	0	0	0	0	0	10, 28	2, 11.8	0	0	12, 17.4
	*bla*_IMP_	0	0	0	0	0	0	0	0	0	0	0	0	0	0	0	0	0	0
	*bla*_KPC_	2, 6.5	0	0	0	2, 6.5	0	0	0	0	0	0	0	0	0	0	0	0	0

HCG, Hospital Civil de Guadalajara “Fray Antonio Alcalde” in Guadalajara, Jalisco; HGM, Hospital General de México “Dr. Eduardo Liceaga” in Mexico City; HGL, Hospital General de León in León, Guanajuato; and HSLP, Hospital Central “Dr. Ignacio Morones Prieto” in San Luis Potosí.

**Table 4:** Resistance phenotypes in Gram-positive ESKAPE isolates from four hospitals in Mexico.

Microorganism	*Staphylococcus aureus*					*Enterococcus faecium*				*Enterococcus faecalis*				
Hospital	HCG (*n* = 86)	HGM (*n* = 82)	HGL (*n* = 26)	HSLP (*n* = 13)	All (207)	HCG (*n* = 26)	HGM (*n* = 3)	HGL (6)	All (*n* = 35)	HCG (*n* = 56)	HGM (*n* = 8)	HGL (*n* = 14)		All (*n* = 78)
Cefoxitina (%)	24.4	53.7	11.6	15.4	35.5	N/A	N/A	N/A	N/A	N/A	N/A		N/A	N/A
Cefinase test (%)	91.7	97.1	85.7	80	87.9									
D-test (%)	22.6	8.9	18.1	0	13.6									
HLAR Test (%)	N/A	N/A	N/A	N/A	N/A	38.5	0	16.6	31.4	30.4	37.5		35.7	32

N/A: Not applicable. HLRA: High-Level Aminoglycoside Resistance. HCG, Hospital Civil de Guadalajara “Fray Antonio Alcalde” in Guadalajara, Jalisco; HGM, Hospital General de México “Dr. Eduardo Liceaga” in Mexico City; HGL, Hospital General de León in León, Guanajuato; and HSLP, Hospital Central “Dr. Ignacio Morones Prieto” in San Luis Potosí.

**Table 5:** Resistance genes in Gram-positive ESKAPE isolates from four hospitals in Mexico.

Microorganism		*Staphylococcus aureus*					*Enterococcus faecium*				*Enterococcus faecalis*			
Hospital		HCG	HGM	HGL	HSLP	All	HCG	HGM	HGL	All	HCG	HGM	HGL	All
Vancomycin resistance	*vanA* (%)	0	0	0	0	0	100	100	100	100	100	0	0	100
β-lactam resistance	*mecA* (%)	61.9	100	66.6	75	88.6	N/A	N/A	N/A	N/A	N/A	N/A	N/A	N/A
	*blaZ (%)*	81.3	84.1	80.7	84.6	98.8	76.19	100	100	80.8	100	0	100	100
Aminoglycoside resistance	*aph(3′)-IIIa* (%)	0	0	0	0	0	0	0	0	0	0	0	0	0
	*ant(4´)-Ia* (%)	0	11	23	0	14.8	0	0	0	0	0	0	0	0
	*acc(6′)-aph(2)* (%)	0	0	0	0	0	90	0	17	62.5	65	67	23	48.5
	*aph(3′)-III* (%)	0	0	0	0	0	90	0	67	81.3	76	67	46	60.6
	*acc(6′)-Ie-aph(2’’)-Ia* (%)	0	89	16	0	37	80	0	17	56.3	65	67	23	48.5
	*aph(2’’)-Ic* (%)	0	0	0	0	0	0	0	0	0	0	0	15	6.0
Macrolide resistance	*ermA* (%)	65.4	86	50	75	78.9	N/D	N/D	N/D	N/D	N/D	N/D	N/D	N/D
	*ermB* (%)	3.9	0	0	0	1.1	80	100	80	81.2	91.7	43	55	77.8
	*ermC* (%)	11.5	2	100	50	11.1	N/D	N/D	N/D	N/D	N/D	N/D	N/D	N/D
	*msrA* (%)	0	4	25	0	3.3	N/D	N/D	N/D	N/D	N/D	N/D	N/D	N/D
Tetracycline resistance	*tetM (%)*	N/A	N/A	N/A	N/A	N/A	100	100	100	100	97	57	72	87.7

N/A: Not applicable. N/D: Not determined. HCG, Hospital Civil de Guadalajara “Fray Antonio Alcalde” in Guadalajara, Jalisco; HGM, Hospital General de México “Dr. Eduardo Liceaga” in Mexico City; HGL, Hospital General de León in León, Guanajuato; and HSLP, Hospital Central “Dr. Ignacio Morones Prieto” in San Luis Potosí.

**Table 6:** Number of ESKAPE Gram-negative isolates carrying coexisting resistance genes from different antibiotic families in four hospitals in Mexico.

Genes	Hospital						
	HCG	HGM	HGL	HSLP	HCG	HGM	HCG
	*K. pneumoniae*				*E. cloacae*		*A. baumannii*
*bla*_CTX-M_ *+ bla*_TEM_	0	2	0	0	1	1	0
*bla*_TEM_ *+ bla*_SHV_	0	0	3	6	0	0	0
*bla*_CTX-M_ *+ bla*_SHV_	19	7	0	0	0	0	0
*bla*_CTX-M_ *+ bla*_SHV_ *+ bla*_TEM_	48	14	0	0	0	0	0
*bla*_NDM_ *+ bla*_CTX-M_ *+ bla*_TEM_	1	0	0	0	0	0	0
*bla*_NDM_ *+ bla*_CTX-M_ *+ bla*_SHV_	5	0	0	0	0	0	0
*bla*_NDM_ *+ bla*_CTX-M_ *+ bla*_SHV_ *+ bla*_TEM_	14	0	0	0	0	0	0
*bla*_KPC_ + *bla*_CTX-M_ + *bla*_SHV +_ *bla*_TEM_	2	0	0	0	0	0	0
*bla*_OXA-23_ + *bla*_TEM_	0	0	0	0	0	0	9
*bla*_OXA-24_ + *bla*_TEM_	0	0	0	0	0	0	18
*bla*_OXA-23_ + *bla*_OXA-24_	0	0	0	0	0	0	1
*bla*_OXA-23_ + *bla*_OXA-24_ + *bla*_TEM_	0	0	0	0	0	0	1

HCG, Hospital Civil de Guadalajara “Fray Antonio Alcalde” in Guadalajara, Jalisco; HGM, Hospital General de México “Dr. Eduardo Liceaga” in Mexico City; HGL, Hospital General de León in León, Guanajuato; and HSLP, Hospital Central “Dr. Ignacio Morones Prieto” in San Luis Potosí.

**Table 7:** Number of *S. aureus* isolates carrying coexisting resistance genes from different antibiotic families in four hospitals in Mexico.

Genes	Hospital			
	HCG	HGM	HGL	HSLP
*blaZ + mecA*	0	1	0	0
*blaZ + mecA + ermA*	11	42	0	2
*blaZ + ermA*	5	0	0	0
*blaZ + ermB*	1	0	0	0
*blaZ + ermC*	2	1	1	1
*blaZ + mecA + ermA + ermC*	0	0	2	1
*mecA + msrA*	0	1	0	0
*blaZ + ermC + msrA*	0	0	1	0
*blaZ* + *aac(6′)-Ie-aph(2″)-Ia* + *ant (4′)-Ia*	0	1	2	0
*blaZ* + *aac(6′)-Ie-aph(2″)-Ia*	0	5	0	0
*blaZ* + *ant(4′)-Ia*	0	0	1	0

HCG, Hospital Civil de Guadalajara “Fray Antonio Alcalde” in Guadalajara, Jalisco; HGM, Hospital General de México “Dr. Eduardo Liceaga” in Mexico City; HGL, Hospital General de León in León, Guanajuato; and HSLP, Hospital Central “Dr. Ignacio Morones Prieto” in San Luis Potosí.

**Table 8:** Number of *E. faecium* and *E. faecalis* isolates carrying coexisting resistance genes from different antibiotic families in four hospitals in Mexico.

Genes	Hospital					
	HCG	HGM	HGL	HCG	HGM	HGL
	*E. faecium*			*E. faecalis*		
*vanA + tetM*	2	0	0	0	0	0
*vanA + tetM + ermB*	2	0	0	0	0	0
*vanA + ermB*	1	0	0	0	0	0
*blaZ + tetM*	1	0	0	0	0	0
*blaZ + tetM + ermB*	5	0	0	1	1	0
*ermB + tetM*	3	1	0	17	1	1
*blaZ + acc(6′)-aph(2)* + *aph(3′)-III + tetM + ermB*	1	0	0	0	0	0
*blaZ + acc(6′)-aph(2)* + *acc(6′)-Ie-aph(2″)-Ia + ermB*	1	0	0	0	0	0
*blaZ + aph(3′)-III + tetM + ermB*	1	0	3	0	0	0
*blaZ + vanA* + *acc(6′)-aph(2)* + *aph(3′)-III* + *acc(6′)-Ie-aph(2″)-Ia + tetM*	1	0	0	0	0	0
*blaZ + vanA + acc(6´)-aph(2)* + *aph(3′)-III* + *acc(6′)-Ie-aph(2″)-Ia + ermB*	0	0	1	1	0	0
*blaZ + vanA + acc(6´)-aph(2)* + *aph(3′)-III* + *acc(6′)-Ie-aph(2″)-Ia + ermB + tetM*	1	0	0	0	0	0
*blaZ + acc(6′)-aph(2)* + *aph(3′)-III* + *acc(6′)-Ie-aph(2″)-Ia + ermB + tetM*	5	0	0	0	0	1
*aph(3′)-III* + *ermB + tetM*	0	0	0	1	0	0
*aph(3′)-III* + *aph(2”)-lc* + *ermB + tetM*	0	0	0	0	0	2
*acc(6′)-aph(2)* + *aph(3′)-III* + *acc(6′)-Ie-aph(2″)-Ia* + *ermB*	0	0	0	0	1	0
*acc(6′)-aph(2)* + *aph(3′)-III* + *acc(6′)-Ie-aph(2″)-Ia* + *tetM*	0	0	0	2	0	0
*acc(6′)-aph(2)* + *aph(3′)-III* + *acc(6′)-Ie-aph(2″)-Ia* + *ermB + tetM*	0	0	0	8	1	2

HCG, Hospital Civil de Guadalajara “Fray Antonio Alcalde” in Guadalajara, Jalisco; HGM, Hospital General de México “Dr. Eduardo Liceaga” in Mexico City; HGL, Hospital General de León in León, Guanajuato; and HSLP, Hospital Central “Dr. Ignacio Morones Prieto” in San Luis Potosí.

## Data Availability

The original data presented in the study are openly available in FigShare at https://doi.org/10.6084/m9.figshare.30401851.
